# Social media exposure, school prevention, and adolescent vaping: a structural equation model-based secondary analysis of the Florida youth tobacco survey

**DOI:** 10.3389/fpubh.2026.1738207

**Published:** 2026-04-09

**Authors:** Martin Lange, Mary Martinasek, Nadine Heckert

**Affiliations:** 1 Department of Fitness & Health, IST University of Applied Sciences, Düsseldorf, Germany; 2 Department of Health Science and Human Performance, The University of Tampa, Tampa, FL, United States

**Keywords:** adolescents, risk perception, school prevention programs, social media, vaping

## Abstract

**Objective:**

Despite recent declines, the use of e-cigarette among adolescents remains the most popular. Two factors influencing vaping behavior are exposure to digital content on social media and school-based prevention programs. Therefore, the aims of this study were to assess the roles of social media use, school prevention programs, and gender on the frequency of vaping and knowledge about its harmfulness.

**Methods:**

This study was conducted as a secondary data analysis based on data from the 2024 ‘Florida Youth Tobacco Survey’. The large cross-sectional sample included 49,839 high school and middle school students. The impact of social media use and prevention programs on the frequency of vaping and knowledge about its harmfulness was analyzed using structural equation modeling.

**Results:**

Social Media Use was positively and statistically significantly associated with Frequency of Vaping and negatively associated with Knowledge about Harmfulness. Prevention Programs were negatively related to Frequency of Vaping and positively associated with Knowledge about Harmfulness. For the Gender variable, a statistically significant effect was observed on Frequency of Vaping, while no significant association was found between Gender and Knowledge about Harmfulness. Females reported a slightly higher use of vaping devices than males. Overall, the model demonstrated an acceptable fit to the data.

**Conclusion:**

These results highlight that both school prevention efforts and social media use exert direct, quantifiable impacts on vaping frequency and knowledge about its harmfulness among adolescents. These findings support the development of a multidimensional framework that unites educational domains, digital literacy, and public health communication to address adolescent vaping behavior.

## 1 Introduction

Electronic cigarettes [also known as e-cigarettes, vaping devices, or electronic nicotine delivery systems (ENDS)] are battery-powered devices that produce aerosols by heating liquids that typically contain flavoring agents, nicotine, and other chemicals (1). In 2024, a significant proportion of adolescents reported using e-cigarettes daily (26.3%), and 38.4% reported using them for at least 20 of the last 30 days (2). Despite a significantly decline in e-cigarette use among middle and high school students (2021: 2.07 million; 2024: 1.75 million), e-cigarettes remained the most popular tobacco product (3). The use of e-cigarettes in recent years has raised concerns about its long-term consequences and potential health risks (4, 5). According to longitudinal research, 45.5% of adolescents engaged in vaping, exceeding national predictions (5). The widespread use of e-cigarettes among adolescents has been linked to various risk factors, such as emotional abuse, substance use, and parental smoking, underscoring the need for early preventive measures (5–7).

While vaping was originally viewed in the context of harm reduction, its rising prevalence among adolescents in the US highlights the need for a more comprehensive understanding of its causes and effects (8). A significant number of adolescents perceive vaping e-cigarettes as less harmful than traditional smoking, resulting in increased consumption rates (9). Other research stated that nearly 19 percent of adolescents believed that e-cigarette vapor consisted solely of water, and 40% perceived vaping ENDS as a smoking cessation tool rather than a potential health concern (10). Such misconceptions emphasize the importance of educating people about vaping-related health risks.

Beyond the increasing prevalence, the normalization of vaping among adolescents is strongly influenced by peer interactions and digital culture (5). Social media platforms such as Snapchat, Facebook, Instagram, and TikTok have become significant factors in youth identity formation, frequently portraying vaping as socially desirable, humorous, and trendy (11–13). Additionally, influencer marketing and social media have reshaped behavioral norms with curated posts and algorithm-driven content, and the risks are often subtly diminished (14, 15). These dynamics highlight the necessity of examining how digital environments interact with individual determinants and institutional health education to shape adolescent vaping behavior (5, 16).

Two factors that are associated with adolescent vaping behaviors have been identified: school-based prevention programs and social media use. However, their respective influences have not been thoroughly examined (15, 17). Social media platforms such as Instagram, TikTok, Facebook, and Snapchat have played a significant role in shaping adolescents’ perception of e-cigarettes, as they are major platforms exposing their users to pro-vaping content (11, 12). A study analyzed the effect of positive and negative portrayals of e-cigarette use on TikTok and found that 63% of the videos depicted e-cigarette use positively and 13% negatively (12). Other platforms, such as Instagram and Snapchat, have also been significantly associated adolescent vaping behaviors by displaying advertisements related to vaping, product placements, and influencer marketing (13, 15).

Misinformation about the harmfulness of vaping is widespread on social media; meanwhile, school-based prevention programs aim to educate students about the health risks associated with vaping, although their effectiveness varies (18, 19). Interventions based on social media have shown mixed results, with some increasing awareness but failing to produce long-term behavioral changes among adolescents (20). Conversely, promising results have been observed in decreasing smoking initiation through school-based prevention programs that teach resistance skills and social interaction confidence, although their effectiveness in preventing vaping remains inconsistent (18, 21). While some interventions increase risk perception and awareness, existing evidence indicates that they do not necessarily inhibit e-cigarette initiation (18).

Social media serves as a platform for both anti- and pro-vaping content, but the dissemination of misinformation may undermine prevention efforts (19). However, variations in student engagement and program implementation may limit the universal effectiveness of school-based prevention initiatives (22), especially in terms of gender differences. These findings suggest that school-based prevention efforts and social media use significantly influence adolescent vaping behaviors, and understanding their relationship is crucial for refining the public health strategies.

Although previous research has explored the role of social media use and prevention programs and their influence on vaping behavior, limited evidence exists regarding their specific effects on adolescents’ knowledge about harmfulness and vaping frequency (15, 18). Consequently, further research is needed to determine the roles of school-based prevention programs, social media use, and gender on knowledge about harmfulness and vaping frequency. Therefore, the aims of this study were to assess the roles of (1) social media use, (2) school-based prevention programs, and (3) gender on adolescents’ frequency of vaping and knowledge about its harmfulness.

## 2 Conceptual framework and hypothesis development

### 2.1 Prevalence and frequency of vaping

In recent years, the prevalence of vaping among adolescents has increased significantly, surpassing national estimates in the US (5). Research has indicated that e-cigarette use, particularly among adolescents, has become a significant public health concern, with prevalence rates exceeding those of conventional cigarette smoking (5, 7). A recent study demonstrated a steady increase in adolescent vaping behavior over the past decade, especially in North America and Europe, revealing a continuous rise in e-cigarette use (23).

Several studies have identified that a substantial number of middle and high school students have experimented with e-cigarettes, indicating widespread accessibility and exposure to vaping (5, 7). One key factor contributing to the initiation of vaping among adolescents is the availability of flavored e-liquids, particularly those with fruit and sweet flavors (24). Research indicates that early exposure to e-cigarettes often leads to long-term use, and that adolescents who experiment with e-cigarettes are more likely to transition into regular users over time (25). Increasing peer influence and social acceptability have also contributed to growing vaping trends (9).

Demographic differences have been observed in the use and continued use of e-cigarettes, with boys being more likely to report current vaping than girls (26). A study found that males exhibited a higher vaping prevalence than females among various age groups, and that males also took more puffs per episode than females (23). These variations in consumption patterns may be influenced by psychosocial factors such as targeted marketing, peer pressure, and risk-taking behavior (7). Similarly, a study demonstrated that e-cigarette use was higher among boys and that the most prominent risk factors were related to parents and peers (27). Collectively, these findings highlight the need for prevention strategies and health education that consider psychosocial factors and gender-specific patterns influencing vaping behavior.

Risk behaviors, including tobacco smoking and alcohol consumption, are closely linked to current e-cigarette use (7). The evidence highlights that vaping is often part of a larger pattern of substance use. Adolescents also engage in cannabis consumption, traditional smoking, and binge drinking (24). A longitudinal study demonstrated that early exposure to nicotine through vaping can act as a gateway to cigarette use and other substances (25). Among adolescent vapers, nicotine was reported as the second most commonly used substance; however, a significant number also indicated a preference for vaping “just flavoring” (24).

Individuals found it challenging to quit or reduce vaping despite recognizing vaping dependence (9). Daily vapers showed a higher nicotine dependency than non-daily vapers, reported stronger cravings, greater difficulty in regulating their intake, and higher consumption rates (25), suggesting that frequent e-cigarette use may lead to a more severe addiction (24). This aligns with evidence that the rapid development of nicotine addiction among adolescents, causing withdrawal symptoms and difficulties in cessation (25). Behavioral cues, such as linking e-cigarette use to emotional states or specific activities, often intensify psychological dependence on vaping (9). Facilitators that promoted e-cigarette use throughout the day included environmental cues, social situations, and stress (9).

### 2.2 Knowledge about its harmfulness

Although some risks are associated with vaping, a significant number of students held misconceptions about the safety and composition of e-cigarettes (10). This study reported that 19.05% of participants believed that e-cigarette vapor consisted only of water, and 23.03% did not categorize e-cigarettes as tobacco products (10). These misperceptions demonstrate a knowledge gap, underscoring the need for educational programs (10). Another study found that about 65% of students stated that the last substance they used was a vape containing “just flavoring,” and that this belief was consistent across different demographic groups (24). Recent evidence suggested that the perception that non-nicotine e-cigarettes are harmless has driven the widespread use of e-cigarettes among adolescents (23). These findings have challenged the common assumption that all vaporizers inhale nicotine, as evidence showed that adolescents may not be fully aware of the contents of e-cigarettes (5).

Beyond misconceptions about the composition of e-cigarettes, public health efforts are further complicated by adolescents’ perceptions of their harmfulness (4). Although concerns regarding harmful chemicals in e-cigarettes exist, adolescents especially perceive vaping as significantly less harmful than smoking traditional cigarettes (4, 10). Additionally, parental education played a significant role and students who perceived e-cigarettes as more harmful were less likely to continue using them (7).

The long-term health effects of e-cigarettes, including their impact on adolescent respiratory health and brain function, have also been evaluated (4). Despite these health concerns, 40.36% of adolescents believed that e-cigarettes were intended for smoking cessation, and 43.13% perceived them as being safer than regular cigarettes (10). These findings underscore the need for public health campaigns and school-based prevention programs that increase awareness about the health risks of vaping (4).

### 2.3 Social media use and prevention programs

Various factors contributed to vaping behaviors, including emotional abuse, parental vaping, and mental health issues, and that adolescents who encountered these were at a greater likelihood of engaging in vaping trajectories (5, 6). Additionally, social media use and school prevention programs play significant roles in shaping vaping behaviors (15, 17, 18). A recent study assessing vaping-related TikTok videos identified the widespread presence of vaping content, including themes such as vaping tricks, comedy, marketing, lifestyle, and nicotine addiction (12). A total of 63% of the digital content depicted e-cigarette use positively, while 13% of the total views were negative (12). Moreover, another study showed that other social media platforms, such as Instagram, Facebook, and Snapchat have emerged as key platforms for influencer marketing and product placement (11, 15), which are significantly correlated with vaping behaviors (8, 28). Instagram content has been analyzed, revealing that advertisements (29%), vaping-product-related posts (28%), and vaping activities (18%) are the most frequent themes (11). These findings demonstrate the effectiveness of influencer marketing and vaping promotions among adolescents in shaping perceptions of e-cigarette use (11). Widespread exposure to vaping-related content on social media is associated with increased susceptibility to vaping among adolescents (22). Among young adult vapers, Snapchat has been identified as the most used platform, with 80 percent of users engaging daily (15). This study also found a connection between vaping uptake and digital engagement (15). Similar trends were observed on Twitter/X, where influencers portrayed vaping as a tool for smoking cessation (28%) and directly promoted vapor products (24%) (13).

Beyond using social media as a marketing tool influencing vaping behavior, engagement with vaping-related online content has been linked to addiction (17). Individuals who interacted with vaping-related online content were more likely to report addiction (17). Those with high sensation-seeking tendencies and those using stronger nicotine concentrations were more likely to develop a susceptibility to compulsive use, underscoring the role of social media in perpetuating vaping behaviors (17). Psychological and social strategies are frequently employed to attract adolescents by displaying vaping content associated with freedom, social acceptance, and fun (14). Understanding these tactics is crucial for creating effective preventive and counter-messaging strategies (14).

School-based prevention programs are regulated in the Florida Statute 381.84, ranging from conter-marketing measures, educational programs, counseling to surveillance and evaluation measures. They play a significant role in shaping adolescent vaping behaviors (18). Several studies have emphasized the need for tailored prevention programs for specific student populations (16, 22, 29). Prior research has shown that anti-vaping messages are seen as overly generic, requiring multiple strategies targeting different levels of vaping behavior (16, 29). One study suggests that younger adolescents may be less responsive to school-based interventions, underscoring the importance of developmentally appropriate strategies (22). Despite these challenges, several interventions have demonstrated potential in reducing susceptibility and influencing behavior (22). Exposure to the US Food and Drug Administration’s (FDA) anti-vaping campaign “The Real Cost” increased negative attitudes toward vaping, lowered intentions to vape, and raised awareness of the dangers of vaping (30). There is limited evidence regarding school-based interventions for preventing initial e-cigarette use; however, such initiatives have been associated with enhanced awareness of harm perception and risk knowledge (18). Ultimately, the widespread dissemination of pro-vaping content on social media platforms, combined with the inadequacies of traditional prevention programs, highlights the need for a comprehensive and multi-faceted intervention strategy.

### 2.4 Relationship of knowledge about harmfulness, vaping frequency, social media, and prevention programs

The increasing prevalence of vaping among young adolescents is a significant public health concern (5). Research has shown that older adolescents tend to vape more frequently, and insufficient knowledge about its harmfulness contributes to their continued use (31). Throughout the day, adolescents often use e-cigarettes habitually, making cessation efforts more difficult and reinforcing nicotine dependence (9). According to the Centers of Disease Control and Prevention (CDC), adolescents are especially susceptible to vaping, as exposure at this stage can harm brain functions, such as attention, making them more vulnerable due to limited knowledge (32).

Social media use exacerbates the vulnerability to vaping by playing a dual role: it can serve as a prevention intervention tool as well as a platform to promote e-cigarettes (19, 30, 33). Several studies have shown that frequent social media use on platforms such as TikTok, Snapchat, and Instagram is associated with increased e-cigarette use, reinforcing the need for targeted prevention and intervention campaigns (11, 12, 15). Social media influencers often portray vaping as an act of defiance against vaping regulations and lifestyle choices, thereby normalizing it among young adolescents (13). Studies have displayed misconceptions about the risks of vaping among students, fueled by misinformation on social media platforms (19, 34). For example, a significant number of tweets (Platform Twitter/X) contained misinformation, either to endorse the unverified health advantages of e-cigarettes or to exaggerate anti-vaping assertions (19). These findings highlight the need for tailored interventions to decrease the spread of misconceptions on social media platforms and the knowledge gap (19, 28, 35). Despite posing certain risks, social media has the potential to be an effective platform for prevention efforts (8). Social media messages significantly increased beliefs and knowledge regarding the harmfulness of e-cigarettes, especially messages about harmful chemicals and lung damage (33). Nevertheless, previous research has stated that social media interventions remain underutilized for vaping cessation efforts (20, 36). Based on these findings, we hypothesize the following:

*H*1: Higher social media engagement is positively associated with the frequency of vaping.

*H*2: Higher social media engagement is negatively associated with knowledge about the harmfulness of vaping.

Another crucial role in shaping adolescent behaviors regarding e-cigarettes play school-based prevention programs (18). While there is evidence that shows the role of school-based prevention programs in adolescent smoking, their effectiveness regarding e-cigarette use remains uncertain (18). Another study stated that interventions increased harm perceptions and vaping risk awareness; however, there is limited evidence on whether they prevent e-cigarette initiation (18, 22). Regarding the prevention of smoking onset, a combination of multiple interventions, including social media literacy training, has proven to be successful and could be adapted for e-cigarette prevention (21, 34). Developmental differences suggest that younger adolescents may be less responsive to generic prevention programs, further highlighting the necessity of tailored prevention programs (22). Additionally, gender differences were observed in vaping behaviors and responses to prevention efforts, underscoring the need for gender-sensitive approaches in developing effective interventions (23, 27). Based on these findings, we hypothesize the following:

*H*3: Higher exposure to school-based prevention programs is negatively associated with vaping frequency.

*H*4: Higher exposure to school-based prevention programs is positively associated with knowledge about vaping-related harmfulness.

*H*5: Gender will exert significant effect on both vaping frequency (*H*5_a_) and knowledge about vaping-related harmfulness (*H*5_b_).

**Figure 1:**
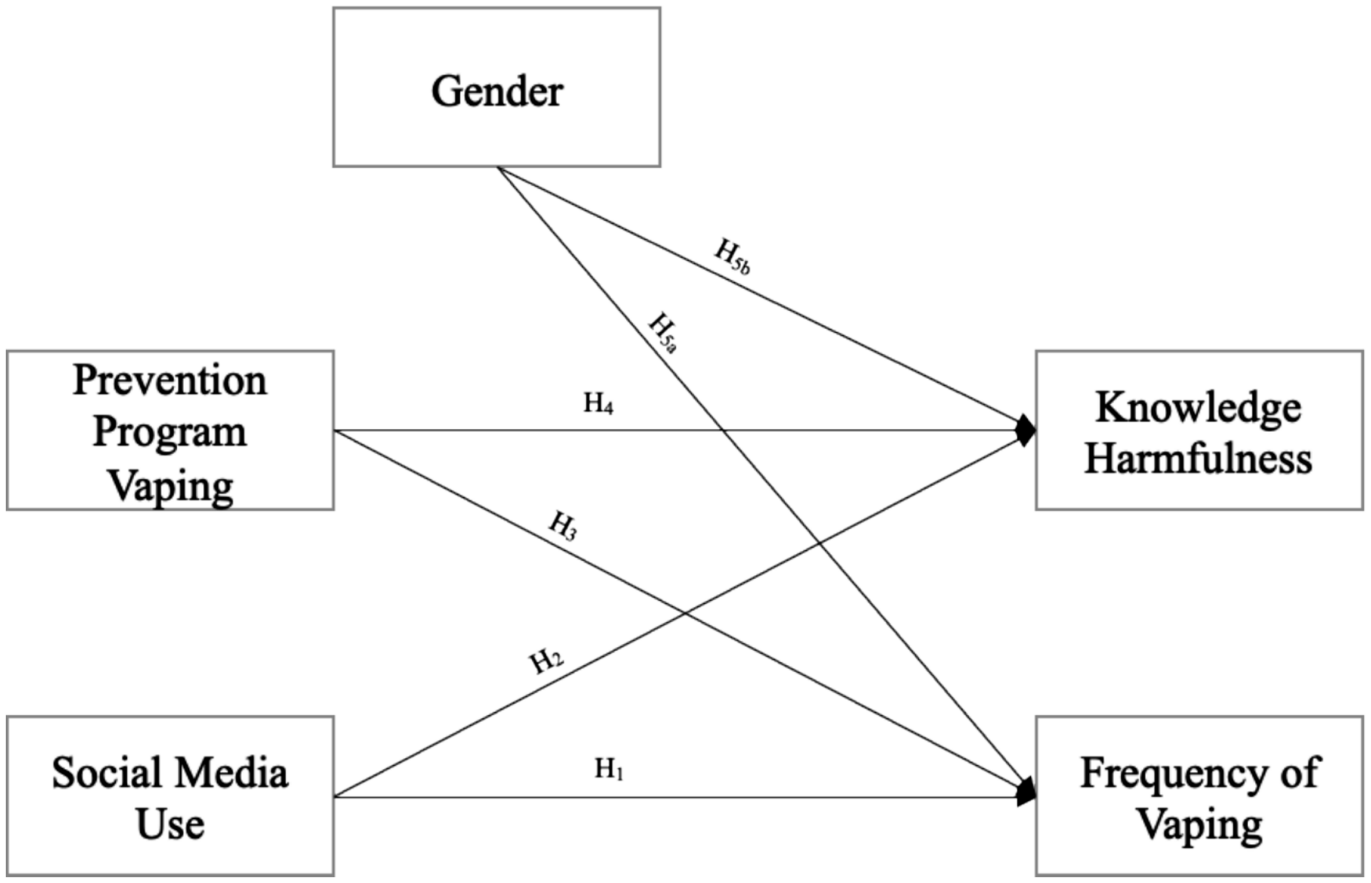
Path diagram of the structural equation model.

Figure 1 summarizes the postulated hypotheses in a hypothetical framework.

## 3 Methodology

### 3.1 Study design

This study was conducted as a secondary analysis using cross-sectional data from the “Florida Youth Tobacco Survey” (FYTS). The FYTS was designed to assess tobacco-related behaviors, influences, and attitudes among adolescents in Florida and is administered by the Florida Department of Health (37). This secondary analysis was approved by the University of Tampa and adhered to the ethical guidelines for human research. The implementation of this study was carried out according to the STROSA-1 standards (Standardized Reporting Routine for Secondary Data Analysis) (38). Reproducibility, transparency, and methodological rigor were ensured by adhering to these standards, which provide standardized reporting criteria for study design, data management, and analytical methodologies (38).

As the primary analytical approach, structural equation modeling (SEM) was used to examine the interdependencies among manifest variables related to adolescent vaping behavior (39, 40). Since most of the included constructs are assessed through manifest variables our model resembles a path model. However, SEM is a more adequate approach, as it allows for the modeling of complex relationships and provides a comprehensive framework for testing direct and indirect pathways, evaluating theoretical assumptions, offering a model fit and controlling for measurement errors (39).

### 3.2 Data flow

The Division of Community Health Promotion at the Florida Department of Health provided data from the FYTS, which was last administered in the spring of 2024 (41). Data are collected annually at the state level and biennially at the county level, ensuring a broad representation of the student population in Florida (37). The FYTS dataset was weighted to reflect the full population of public high and middle schools in Florida, thereby increasing the generalizability and applicability of the findings (41). Data access and security protocols were developed in collaboration with the University of Tampa and Florida Department of Health. The FYTS also adheres to ethical guidelines, with all responses anonymized to ensure participant confidentiality (37).

### 3.3 Units of analysis

The cross-sectional survey participants consisted of individual middle and high school students aged 9 to 21 residing in Florida (41). In the 2024 administration of the FYTS, a total of 50,296 students participated: 25,293 at the middle school level and 25,003 at the high school level (41). Data were collected from 60 Florida counties, and 652 public middle and high schools participated in the FYTS (41). Stratified random sampling ensured proportional representation across demographic characteristics, including gender, race/ethnicity, grade level, and geographic location. The FYTS was voluntary and self-administered using an anonymous, standardized questionnaire completed by high school and middle school students in classroom settings after parental consent was obtained (37).

**Figure 2:**
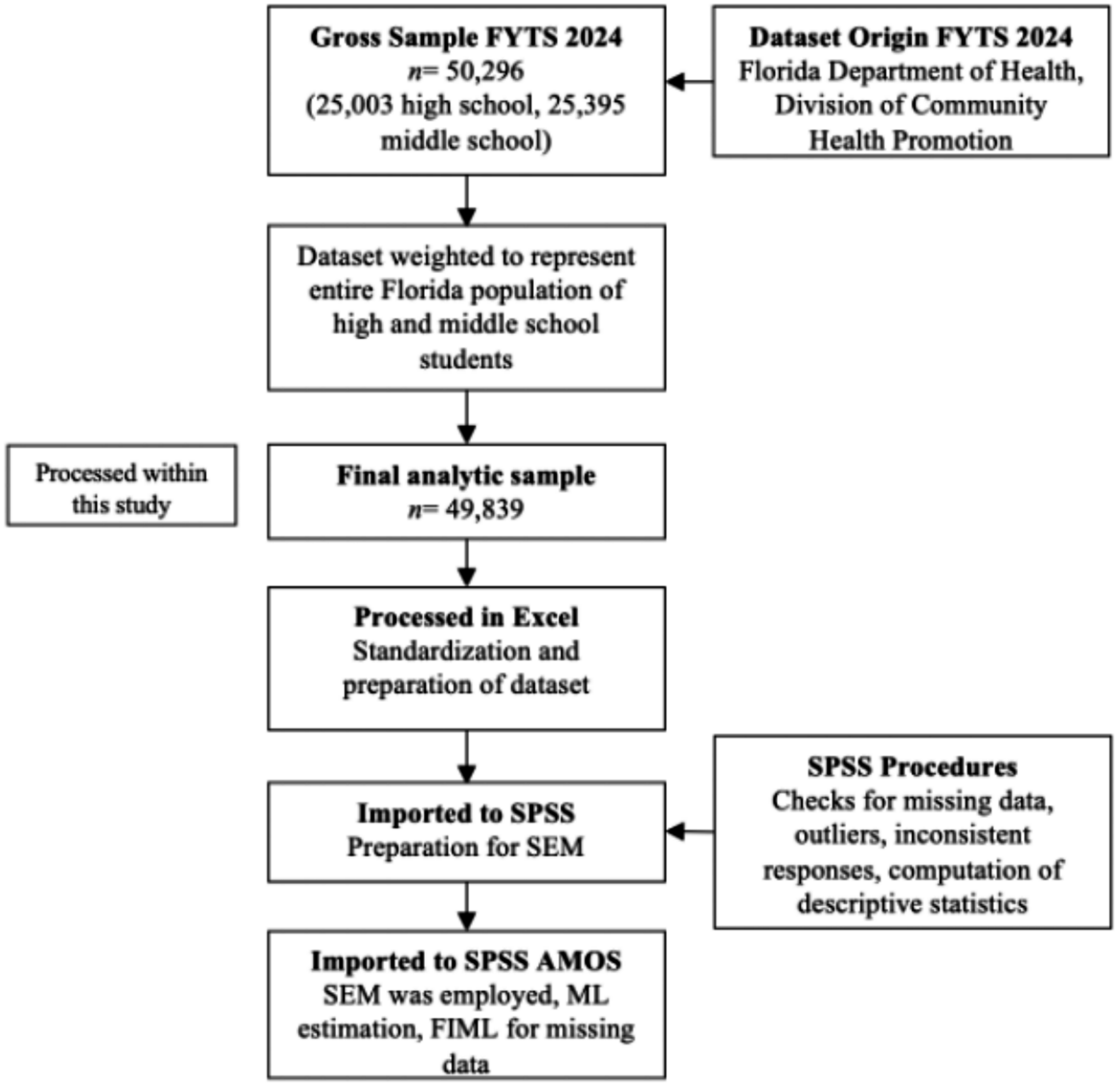
Flowchart of selection process from original to study population.

The final analytic sample included *n* = 49,839, providing an adequate number of observations per variable to achieve sufficient statistical power in the SEM. The dataset was subjected to rigorous cleaning procedures, including checks for missing data, inconsistent responses, and outliers, thereby optimizing data quality for subsequent analyses. The entire sampling and selection process is shown in Figure 2.

### 3.4 Variables and model specification

The five variables in this study were carefully chosen to address the research questions and hypotheses regarding vaping behaviors, knowledge about harmfulness, gender, and the roles of social media use and prevention programs. SEM was employed to examine the relationship between the dependent and independent variables and to explore the direct effects within the model.

*Gender* was measured as a nominal independent variable and categorized dichotomously. The variable *Prevention Program Vaping* was defined as a nominal independent variable, indicating whether schools provided programs or resources to quit vaping. *Social Media Use* was operationalized as an ordinal independent variable that captured the frequency of interaction with the Facebook app. Other social media concepts (e.g., Snapchat, TikTok) could not be included due to unstable parameter estimates and poor model fit in the preliminary analyses. Since Facebook is one of the four most widely used social media platforms adolescents, the variable Facebook was used to represent the concept *Social Media Use* (42–45). *Knowledge Harmfulness* was defined as a nominal dependent variable that captured whether participants believed that using electronic vapor products was harmful to their health. *Frequency of Vaping* was treated as an ordinal, dependent variable, indicating how many days participants used an electronic vapor product during the past 30 days (see Table 1). All variables were standardized using Microsoft Excel prior to conducting the SEM analysis. Standardization was crucial for eliminating potential biases and allowing meaningful comparisons.

**Table 1:** Measurement properties of the study variables.

Variable	Abbreviation	Level of measurement	Scale range	Original item in the FYTS
Dependent				
Knowledge harmfulness	KH	nominal	0-2	17
Frequency of vaping	FV	ordinal	0-6	21
Independent				
Prevention program vaping	PP	nominal	0-2	48D
Social media use	SM	ordinal	0-6	92A
Gender	G	nominal	0-1	2

Regarding the model specification we estimated the residual covariance between the two endogenous variables to account for shared unexplained variance. Covariances among exogenous variables were constrained to zero.

### 3.5 Statistical analysis

SEM was employed to examine the relationships between variables, such as social media use and vaping behavior. All variables were operationalized using single-item measures, and descriptive statistics (*n*, minimum, maximum, mean, standard deviation [SD]) were computed for all variables (39, 40). Data preparation was conducted using Microsoft Excel, and the cleaned and formatted dataset was used for further analysis. Continuous variables were standardized, and categorical variables were coded as dummy variables. Standardizing the path coefficients allowed for interpretation in standardized units, thereby improving the interpretability of the statistical relationships (40).

Model estimation was performed using the lavaan package (Version 0.6-19;) in R (Version 4.x) under a recursive framework (46, 47). The model incorporated direct paths from independent variables to dependent outcomes. Due to the binary and ordinal nature of some variables the diagonally weighted least squares (DWLS) approach was used as an estimation method (48, 49), also known as Weighted Least Squares Mean and Variance Adjusted (WLSMV) in lavaan. WLSMV works well for sample sizes over 200 and is known to outperform maximum likelihood (ML) estimates in nearly all asymmetric data conditions (49–51). It is also robust fo binary and ordinal variables with few categories (52).

Model fit was evaluated using multiple indices, including Chi-square test (𝜒^2^), Tucker-Lewis Index (TLI) Root Mean Square Error of Approximation (RMSEA), Standardized Root Mean Square Residual (SRMR) and Comparative Fit Index (CFI) (53, 54). To assess the robustness of the findings, a sensitivity analysis was conducted using maximum likelihood estimation with Full Information Maximum Likelihood (ML/FIML). This analysis served to evaluate the stability of parameter estimates and substantive conclusions under an alternative estimation approach that explicitly accounts for missing data under the assumption of missing at random. The ML/FIML analysis utilized *n* = 49,781 observations with 28 missing data patterns; empty cases (*n* = 58) were excluded (55).

The primary WLSMV models were estimated using the same effective sample size, with pairwise deletion applied for missing values on ordered variables. Standardized regression weights (*β*) were reported to reflect the strength of associations, allowing for direct comparison of effect sizes across predictor variables. Statistical inference was based on standardized direct effects, with significance determined using *p*-values and critical ratios (40). Reporting followed established guidelines for structural equation modeling to ensure transparency and reproducibility (56).

## 4 Results

### 4.1 Descriptive statistics

Descriptive statistics were calculated for each of the five observed variables: gender, exposure to prevention programs on vaping, social media engagement on Facebook, frequency of vaping, and knowledge about vaping-related harmfulness. Table 2 presents the descriptive statistics of the study variables.

**Table 2:** Descriptive statistics.

Item response options	N	Coding	%
Gender	49,327		
Female	24,302	0	48.80
Male	25,025	1	50.20
Knowledge Harmfulness	49,081		
Yes	3,298	0	6.60
No	40,861	1	82.00
Not Sure	4,922	2	9.90
Frequency of Vaping	48,759		
0 days	44,447	0	89.20
1 or 2 days	1,478	1	3.00
3 to 5 days	531	2	1.10
6 to 9 days	361	3	0.70
10 to 19 days	435	4	0.90
20 to 29 days	304	5	0.60
All 30 days	1,203	6	2.50
Prevention Program Vaping	46,585		
Yes	9,444	0	20.30
No	10,100	1	21.70
Not Sure	27,041	2	58.00
Social Media Use	41,375		
Never	26,853	0	64.90
Every few months	3,581	1	8.70
Every few weeks	2,485	2	6.00
1-2 days a week	1995	3	4.80
3-5 days a week	1,052	4	2.50
Once a day	2058	5	5.00
Several times a day	3,351	6	8.10

The gender variable was reported by 49,237 respondents and the mean value of 0.51 (*SD* = 0.50) stated an approximately equal distribution of male and female participants within the sample. Knowledge about the harmfulness of vaping was assessed based on 49,081 valid responses and the mean score was 1.03 (*SD* = 0.41). Regarding the frequency of vaping, the mean score was measured at 0.29 (*SD* = 1.12) among the 48,759 participants. Exposure to school-based prevention programs regarding vaping was assessed based on 46,585 participants, and the mean score was 1.38 (*SD* = 0.80). The engagement with the social media app Facebook was reported by 41,375 participants and the mean score was 1.19 (*SD* = 1.98).

### 4.2 Model fit

Since the WLSMV estimator is a scaled test for categorical data and nonnormality and therefore behaves differently from ML tests for continuous data, we triangulated model fit by evaluating a range of goodness-of-fit indices in accordance with established SEM reporting standards (56). The chi-square statistic yielded a significant result (𝜒^2^ = 6.019, df = 1, *p* < 0.014), which is commonly observed in large samples (*n* = 49,781) due to the known sensitivity of the chi-square test to sample size (54). The chi-square to degrees of freedom (df) ratio (𝜒^2^/df = 6.019) exceeded conventional thresholds (> 5). The WLSMV scaling alters the test’s sampling distribution, making the χ^2^/df ratio less stable and less comparable to conventional cutoffs (54). Most additional fit indices, however, surpassed commonly recommended thresholds for good fit (CFI, SRMR, TLI) (47). The RMSEA was 0.010, with a 90 percent confidence interval from 0.005 to 0.013], indicating a marginal fit (54). Additionally, a sensitivity analysis comparing ML-estimation with WLSMV confirmed the robustness of the proposed model (Frequency of Vaping: R^2^_ML_ = 0.021, R^2^_WLSMV_ = 0.095; Knowledge of Harmfulness: R^2^_ML_ = 0.006, R^2^_WLSMV_ = 0.016). Klicken oder tippen Sie hier, um Text einzugeben. Given the performance of all indicators and the sensitivity analysis in both estimations, the model demonstrates adequate fit for the research purpose (54, 57) (see Table 3).

**Table 3:** Model fit.

Model	𝜒^2^	df	𝜒^2^/df	*p*	TLI	CFI	SRMR	RMSEA
Hypothetical model	6.019	1	6.019	0.014	0.963	0.996	0.000	0.011

df, degrees of freedom; TLI, Tucker-Lewis Index; CFI, Comparative Fit Index; RMSEA, Root Mean Squared Error of Approximation; SRMR, Standardized Root Mean Square Residual.

### 4.3 Model specification

The SEM was formulated as a recursive path model. In alignment with the study’s theoretical framework, both the observed exogenous and endogenous constructs were integrated. The observed endogenous variables included knowledge about harmfulness and frequency of vaping. Both endogenous variables were linked to an unobserved exogenous variable (error term), enabling the modeling of residual variance (40). The observed exogenous variables comprised gender, school-based prevention programs for vaping, and social media use operationalized through engagement with the app Facebook.

Directional paths were specified *a priori* based on the empirical literature and established theoretical frameworks. The model was constructed and estimated using lavaan, with standardized estimates employed to facilitate comparability across the paths and enhance interpretability (40). The model was composed of directly observed variables and did not include latent variables; therefore, a confirmatory factor analysis (CFA) was not performed. Each variable was measured as a manifest indicator, and the lack of latent variables is consistent with the nature of the study’s primarily predictive and explanatory objectives (39, 40).

Preliminary descriptive analysis confirmed that all variables demonstrated acceptable distributions with no substantial skewness or kurtosis (40). Prior to the SEM estimation, the missing data patterns were evaluated. Little’s MCAR test was statistically significant (𝜒^2^ = 322.84, *p* < 0.001), suggesting data were not missing completely at random (MCAR) (58).

### 4.4 Structural model estimates

This section presents path estimates derived from the structural equation model, including standardized regression weights, critical ratios, and significance values. These estimates provide an understanding of the direction and strength of the associations between the exogenous and endogenous variables of the model.

The standardized regression weights, standard errors, critical ratios (C. R.), and *p*-values are shown in Table 4 for each hypothesized path. Several statistically significant associations were observed. *Prevention Program Vaping* exhibited a positive and significant effect on *Knowledge Harmfulness* (*β* = 115, *p* < 0.001). *Prevention Program Vaping* was negatively associated with *Frequency of Vaping* (*β* = −0.129, *p* < 0.001). *Social Media Use* was positively associated with *Frequency of Vaping* (*β* = 0.256, *p* < 0.001) and negatively associated with *Knowledge Harmfulness* (*β* = −0.024, *p* < 0.001). Therefore, Hypothesis 1 through Hypothesis 4 were fully supported by statistically significant path estimates.

**Table 4:** Regression weights.

Hypothesized relationships	Standardized estimates	S. E.	C. R.	*p-*values	Hypothesis supported
H1: SM ^+^_→_FV	0.256	0.010	25.185	<0.001	supported
H2: SM ^–^_→_ KH	−0.024	0.008	−3.190	<0.001	supported
H3: PP ^–^_→_ FV	−0.129	0.008	−11.525	<0.001	supported
H4: PP ^+^_→_ KH	0.115	0.007	16.169	<0.001	supported
H5_a_: G _→_ FV	−0.082	0.010	−7.366	<0.001	supported
H5_b_: G _→_ KH	0.007	0.010	0.794	0.427	Not supported

No statistically significant effect was found for *Gender* on *Knowledge Harmfulness* (H5b: *β* = 0.007, *p* = 0.427). However, a significant negative association between *Gender* on *Frequency of Vaping* was found (H5a: *β* = −0.082, *p* < 0.001), demonstrating comparable levels of awareness regarding vaping-related harm, but a difference in vaping frequency between male and female adolescents. Therefore, Hypothesis 5 was partially supported. Additionally, the covariance between the error terms for *Frequency of Vaping* (e2) and *Knowledge Harmfulness* (e1) was statistically significant (*b* = −0.044, *SE* = 0.005, C. R. = −11.42, *p* < 0.001).

Squared multiple correlations (R^2^) indicated that the model accounted for 11.7 percent of the variance in vaping frequency and 2.1 percent in perceived knowledge about harmfulness. Additionally, the modification indices were reviewed to assess potential model improvements, but no modifications were implemented. Alignment with the study’s conceptual framework and commitment to theoretical rigor was prioritized; therefore, the original model structure was retained. Model parsimony and construct validity were prioritized over refinements that were solely driven by statistical optimization.

## 5 Discussion

### 5.1 Main results

A principal result was the positive and statistically significant relationship between *Social Media Use* and *Frequency of Vaping* (*β* = 0.256, *p* < 0.001). In parallel, *Social Media Use* demonstrated a negative association with *Knowledge Harmfulness* (*β* = −0.024, *p* < 0.001). In contrast, *Prevention Program Vaping* exhibited a dual effect: it was negatively related to *Frequency of Vaping* (*β* = −0.129, *p* < 0.001) and positively associated with *Knowledge Harmfulness* (*β* = 0.115, *p* < 0.001). For the *Gender* variable, a statistically significant effect was observed on *Frequency of Vaping* (*β* = –0.082, *p* < 0.001), while no statistically significant association was found between *Gender* and *Knowledge Harmfulness* (*p* = 0.427). Finally, the structural equation model demonstrated an overall acceptable fit to the data with small to modest effects.

### 5.2 Limitations

The present study presents valuable insights into adolescent vaping behavior; however, several methodological limitations should be addressed and carefully considered. First, the use of cross-sectional data prohibits any inference of causality (59). Associations between predictor and outcome variables are correlational in nature, limiting the ability to draw conclusions regarding the directionality of the effects and temporal precedence. Further research utilizing longitudinal data or experimental designs is essential to establish causality and capture dynamic patterns over time.

Furthermore, no latent variables were included in the SEM. Therefore, the ability to model abstract psychological constructs was restricted, as was the ability to account for measurement errors (60). All constructs within the model were operationalized as observed variables, which may have led to an underestimation of the strength of the relationships due to attenuation resulting from measurement imprecision.

Additionally, no mediation or moderating mechanisms were incorporated into the model, which may exclude key explanatory pathways through which predictors influence outcome variables. Future research should explore the role of potential mediators or moderators to gain a more refined understanding of the observed associations. Although the model accounted for several theoretically relevant predictors, it did not include significant factors such as socioeconomic status or peer influence, which may explain the additional variance in the frequency of vaping and knowledge about vaping-related harm. Especially, the “knowledge of harmfulness” needs to be interpreted with caution as the explanatory power of the model’s predictors remains small.

Moreover, the FYTS relies solely on self-reported data, and therefore introduces potential sources of bias, including recall inaccuracies and social desirability bias. Participants may have overestimated their knowledge about vaping-related health risks, or underreported their vaping behavior.

Another methodological limitation concerns the potential influence of algorithmic bias on adolescents’ exposure to digital content presented on social media. Digital platforms such as Facebook, YouTube, Instagram, and TikTok employ sophisticated recommendation algorithms that curate content based on behavioral patterns and user engagement, potentially increasing exposure to pro-vaping content within specific user groups (61). Individualized digital environments are created by these algorithm-driven ecosystems, which are difficult to control or replicate in survey-based research, thereby affecting the ecological validity of the study (62). Research also shows that adolescents often underestimate the persuasive intent embedded in algorithmically delivered content, especially when the digital content is framed as entertainment or is generated by influencers (63).

Several key measures, such as social media use and exposure to school-based prevention programs, were evaluated using a single-item indicator, indicating limited operationalization and accuracy of key constructs. Given the complexity of these constructs, single observed indicators may fail to capture important dimensions, such as the context of use, and reduce validity and reliability, which is a notable limitation. Therefore, our SEM merely provides a basis for further research areas. Reliance on a single social media platform (Facebook) to represent social media use may not adequately reflect the diversity of adolescents’ online activity. Contemporary adolescent populations exhibit higher engagement rates on platforms such as Snapchat, Instagram, and TikTok, which differ substantially from Facebook in terms of user demographics, content format, and algorithmic design (12, 13). Limiting the analysis to a single social media platform may underestimate the broader digital influence on vaping-related behaviors, particularly given TikTok’s popularity among adolescents (15). Nevertheless, Facebook is still a popular platform in general and specifically for young adults with user rates ranging from 51 to 74% that can be seen as a relevant indicator (42–45). Consequently, the lack of measurement scale may reduce reliability, and the multidimensional nature of constructs such as social media use and school-based prevention efforts may not be fully captured. Additionally, while statistically significant associations were found, the effect sizes were generally small and should therefore be interpreted with caution.

In summary, this study provides valuable empirical evidence, but its limitations should guide directions when shaping future research and be considered when interpreting its findings.

### 5.3 Strengths

This study has several conceptual and methodological strengths that enhance the robustness, generalizability, and credibility of its results. First, our model was grounded in an established theoretical framework, which led to a coherent specification of the hypothesized pathways in the study. Theoretical reasoning and empirical evidence were integrated to inform all directional relationships, which were proposed *a priori* to ensure a theory-driven analytical approach (39, 40). Second, the large and demographically representative sample was a key strength of this study, enhancing the generalizability of the findings to a broader adolescent population. The sample’s diversity and scale enabled stable parameter estimation and adequate statistical power to detect subtle or low-magnitude effect sizes in SEM (40, 56). Third, the use of SEM offers a significant methodological advantage, as it allows for the simultaneous estimation of multiple interrelated pathways and representation of complex relationships among variables (40). This method also accounts for measurement errors, which enhances the prevision of the estimated parameters (40). Moreover, applying the SEM enhanced the analytical depth of the study, despite the exclusion of latent constructs from the model (40).

Finally, the SEM demonstrated a robust fit to the observed data, which exceeded the commonly established thresholds (e.g., CFI, RSMEA) (54). These results suggest that the hypothesized model adequately captured the underlying data structure and validity of both the measurement approach and conceptual framework. Collectively, these strengths highlight that the study provides a theoretically coherent and methodologically rigorous contribution to the literature on vaping behavior of adolescents, particularly focusing on the roles of digital determinants and institutional prevention programs on vaping behavior.

### 5.4 Interpretation

The present study examined the role of social media use and school-based prevention programs on adolescent vaping behavior and the knowledge concerning the harmfulness of vaping. Statistically significant and meaningful associations were identified, contributing to a broader understanding of how institutional and digital contexts are associated with adolescent health outcomes. Even though the overall effects are modest, the study’s findings reinforce existing research, offering insights into prevention policy, health communication, and behavioral science. Within this cross-sectional approach these findings serve as important indicicators for future research.

Descriptive statistics provide a foundational overview of the key variables, study population, and demographic composition. The distribution of *Gender* was nearly equal (*M* = 0.51, *SD* = 0.50), indicating that both male and female participants were almost equally represented, supporting the generalizability of the findings across gender categories. Regarding *Knowledge Harmfulness*, the mean score was 1.03 (*SD* = 0.41), suggesting a moderate level of awareness among respondents. However, this also indicates that informational gaps regarding the health risks associated with vaping are still present (4, 10). The average vaping frequency was relatively low (*M* = 0.29, *SD* = 1.12), indicating limited use of electronic vapor products within the adolescent population. Exposure to school-based prevention efforts demonstrated a moderate level (*M* = 1.38, *SD* = 0.80), stating that preventative resources were provided for the student population; however, there may be variability in the extent of implementation. Social media use via Facebook reflected notable heterogeneity in user behavior (*M* = 1.19, *SD* = 1.98), but average utilization among participants remained relatively low. Collectively, these findings suggest that participants were moderately aware of vaping-related health risks, limited vaping behavior, and had intermediate exposure to school-based prevention programs.

Consistent with *H_1_*, a direct effect in the model was observed between *Social Media Use* and *Frequency of Vaping*. These results align with previous research, indicating that adolescents frequently exposed to vaping-related content online are at an increased risk of initiating or engaging in vaping behavior (12, 28). Social media platforms such as Snapchat, Instagram, and TikTok have been widely documented as channels that expose users to pro-vaping content, where vaping is often portrayed as entertaining, socially rewarding, and low-risk (11, 14). Messaging of this nature may reinforce behavioral norms that support habitual use and attenuate the perceived health risks of vaping. Empirical evidence demonstrates that recurrent exposure to vaping-related content on social media platforms is positively associated with increased initiation of e-cigarette use among adolescents, as well as with shifts in their perceptions of acceptability and social norms (64). By amplifying descriptive normative beliefs and facilitating interpersonal communication centered on e-cigarette use, media scanning has directly and indirectly increased the risk of vaping (65). Collectively, the present findings substantiate that digital exposure is not merely passive or incidental but may play a substantial role in reinforcing or escalating health-compromising behaviors.

Concurrently, *Social Media Use* was negatively associated with *Knowledge Harmfulness*, supporting *H_2_*. This inverse relationship suggests a potential decline in health-related awareness when adolescents are exposed to unregulated or misleading online content. These results corroborate existing concerns regarding the informational hazards of social media environments and the normalization of vaping online (19, 66). Adolescents may encounter peer-generated content, understatements of risks, and misinformation online that minimize harm, contributing to inaccurate risk perceptions (19). Therefore, social media serves a dual role as both a behavioral catalyst and a disruptor of accurate information, and is especially preeminent in this context, calling for specific intervention strategies that extend traditional awareness-raising approaches (20, 34). Evidence suggests that such strategies should comprise evidence-based interventions targeting both behavioral and cognitive determinants of health (4, 22).

At the same time, additional factors such as algorithmic amplification, influencer marketing, and peer normalization should be considered, as the mechanisms underlying the impact of social media are complex and their interdependencies are not captured in the present model with its low explanatory power. Prior research indicates that algorithmic amplification can increase selective exposure to engaging pro-vaping content while de-emphasizing harm-related information (67–69). Influencer marketing represents a distinct credibility and framing mechanism, as sponsored or aspirational endorsements may normalize vaping and attenuate risk perception (70, 71). Peer normalization processes further contribute through social learning and descriptive norms, whereby observing peers’ behavior may reduce stigma and shift attention away from potential harms (72, 73). Taken together, these findings suggest that heterogeneous pathways and mediating mechanisms are conflated in a simple direct-effects model, underscoring the need for future research to disaggregate social media exposure by source and content and to incorporate mediating or multilevel analytic approaches.

In contrast, exposure to school-based prevention programs was associated with increased knowledge about the harmfulness of vaping and reduced vaping frequency, as hypothesized in *H_3_* and *H_4_*. These findings align with longstanding evidence that educational interventions can positively impact both behavior and cognition, especially when incorporated with tailored and interactive designs (18, 21). Despite contemporary critiques that many anti-vaping programs lack student engagement, real-world applicability, or credibility, protective associations were still evident (29). Recent findings indicate that eHealth components and peer-led approaches within school-based vaping prevention initiatives can shift attitudes, enhance harm-related knowledge, and decrease intentions to initiate e-cigarette use (74). Programs incorporating theoretical grounding, teacher involvement, and social skills development appear particularly promising; however, inconsistencies across study designs and outcome measures highlight the necessity for more robust longitudinal research designs (74). The findings of the present study underscore the continued potential of prevention programs as a crucial protective factor, especially when supplemented with youth-relevant messaging and tailored formats. These results underscore the ongoing relevance of institutional prevention programs and advocate their strategic enhancement rather than abandonment (18, 22).

A small yet statistically significant effect of *Gender* on *Frequency of Vaping* was identified, indicating that female adolescents reported slightly higher frequencies of vaping than males. This finding contrasts with prior research but substantiates emerging evidence advocating that gender differences in vaping behavior may be shifting (23, 26). Evidence suggests individual factors such as higher levels of sadness, stress and hopelessness as potential contributors, since these shifts have been observed since the Covid-19 pandemic (75). Also, personality and social factors such as sensation seeking or peer influence contribute to the higher rate of female vapers among adolescents in the last years (76). Notably, *Gender* was not significantly associated with *Knowledge Harmfulness*, suggesting that information dissemination efforts are comparably effective across genders in terms of awareness. Therefore, only *H_5a_* was supported. Nonetheless, disparities in behavior remain, potentially reflecting gender-specific influences, such as differential responsiveness to intervention formats or peer dynamics.

Collectively, the findings of this study reinforce the importance of addressing both institutional settings and digital environments in prevention efforts to reduce adolescent vaping behavior. School-based prevention efforts have demonstrated protective potential but may be constrained by competing curricular demands, implementation variability, or limited program duration (22). Simultaneously, the influence of social media use remains insufficiently mitigated and largely unregulated, driven by commercial interests and algorithmic personalization, which may steadily increase exposure to high-risk content over time (20, 77). This duality highlights the necessity of harmonized prevention efforts that span both online and offline environments.

Our findings offer valuable insights for policymakers, public health practitioners, and educators. Moving forward, efforts to mitigate adolescent vaping behavior may be most strongly associated with intervention approaches that are tailored to both school settings and digital environments. Utilizing social media as a platform for positive engagement while reinforcing skills-based prevention through education may enable normative change, ultimately decreasing the prevalence of adolescent vaping and enhancing health literacy.

### 5.5 Transferability

Several key methodological features strengthen the generalizability of the present study’s findings while also acknowledging several important considerations. First, the results are based on data from a large, demographically diverse sample, offering a empirical foundation for generalizing the results to comparable adolescent populations. The large sample size ensured high statistical power and strengthened the stability of the parameter estimates, enabling the detection of small effects with a high level of precision (40). This degree of statistical reliability contributes to the replicability and credibility of the study’s findings while mitigating concerns regarding model instability and sampling variability (40).

Moreover, the context of the data collection also facilitates the external validity of the results. The inclusion of relevant sociocultural predictors, such as social media engagement and exposure to school-based prevention programs, enhances the model’s ecological validity. The contextual relevance strengthens the confidence that the observed associations reflect real-world dynamics rather than statistical artifacts (78).

However, several limitations warrant consideration when evaluating the generalizability of the findings. First, the cross-sectional design restricts inferences to a single time point, thereby constraining conclusions about neither the evolution of the observed relationships (59) nor their causality. Consequently, the results cannot be interpreted as evidence of longitudinal change. Data were collected within a particular educational and national setting, meaning that the observed associations may have been shaped by culturally specific factors, such as public health policies, local norms, and school-based health programs that do not apply in other geographic contexts. Adolescents in other states or countries may be subject to different policy environments, sociocultural influences, and institutional frameworks that could moderate the associations between the predictor and outcome variables in this study.

Also, the generalizability of the present findings should be considered in light of Florida’s specific policy environment regarding vaping and school-based prevention. Florida enforces statewide age restrictions and school campus prohibitions on vaping products (79), and substance use prevention is embedded within mandated health education frameworks. However, the requirements for vaping-specific content and the integration of media literacy into prevention programming remain relatively broad, leaving substantial discretion to school districts and individual schools in terms of implementation (80). Consequently, adolescents’ exposure to formal prevention programs and their alignment with digital media education may vary considerably within the state. These contextual characteristics may limit the direct transferability of the findings to jurisdictions with more centralized, comprehensive, or explicitly regulated vaping prevention and media literacy policies (81).

### 5.6 Practical implications

While the statistical findings of this study are modest, the overall confirmed model points to several practical implications for public health initiatives, educational policies, and digital health communication. In particular, the positive association between *Social Media Use* and increased *Frequency of Vaping* highlights the pressing need for targeted interventions that address the role of digital environments on the health behaviors of adolescents. Adolescents are frequently exposed to digital content that may normalize vaping, and such exposure appears to be linked to increased levels of vaping (78). Accordingly, digital public health strategies should be designed to counter pro-vaping narratives and deliver relatable health information specific to this target group via online platforms (22). At the same time, the observed negative association between *Social Media Use* and *Knowledge Harmfulness* implies that passive exposure to information is inadequate and may misinform under certain circumstances, underlining the need for the incorporation of media literacy education within school curricula. Schools might encourage students to examine how social media can distort perceptions of vaping prevalence and social acceptance through selective visibility and peer signaling. In this way, media literacy components could be explicitly linked to norm-corrective elements of prevention programming. Additionally, students can collaboratively develop counter-messages, critiques, or alternative narratives addressing vaping-related social media content. Such formats may reinforce prevention messages while fostering agency, reflexivity, and resistance to persuasive digital influence. Overall, adolescents need to acquire critical appraisal skills to distinguish credible online content from persuasive or deceptive content (27).

Furthermore, these findings suggest that evidence-based and structured health education within formal educational settings are of relevance. Expanding both the reach and intensity of prevention programs that on the one hand take gender differences into account and on the other hand engage students through active learning, such as scenario-based learning and peer-led discussions, may enhance their effectiveness and reinforce broader public health goals (29, 60).

A multidisciplinary approach involving schools, parents, health departments, and technology companies may facilitate the development of cohesive intervention strategies (29). For instance, strategic partnerships between social media platforms and state educational authorities could enhance the placement of informative content through algorithm-driven content feeds (12). Additional initiatives, such as school-based peer mentorship programs and parental engagement, may help mitigate peer influence and reinforce health-promoting norms (27). Furthermore, the addition of vaping-specific prevention modules to health education curricula ensures both scalability and uniformity across varying regions (22). Collectively, these implications underscore the importance of coordinated efforts across technological, educational, and policy domains to effectively promote the health of adolescents.

## 6 Conclusion

The present study provides an empirically grounded contribution and was designed to examine the role of social media use and school-based prevention programs in adolescent vaping behavior, employing an SEM approach. The model yielded four principal findings based on data from nearly 50,000 participants. First, *Social Media Use* was positively associated with *Frequency of Vaping* and inversely associated with *Knowledge Harmfulness*. Second, exposure to school-based *Prevention Programs* was positively associated with *Knowledge Harmfulness* and negatively associated with *Frequency of Vaping*. Third, *Gender* was found to have a significant effect on vaping frequency, with female adolescents reporting slightly higher use than males, while no gender-based differences emerged in the knowledge about vaping-related harm.

Our findings support existing evidence while simultaneously laying out new findings that are of practical relevance. Linear mediation models that conceptualize knowledge acquisition as the principal mechanism of behavior change are challenged by the observed direct association between social media use and frequency of vaping. Instead, the findings suggest that algorithmically tailored content, social norms, and affective cues embedded within digital platforms may bypass or potentially diminish risk awareness, thereby intensifying vaping behavior. Simultaneously, the positive association of school-based prevention programs with both behavioral and cognitive outcomes underscores the relevance of interactive, skills-based educational approaches in schools. These findings highlight the necessity of enhancing delivery methods, program content, and student engagement to counterbalance the pervasive associations observed between digital media exposure and adolescent vaping.

In conclusion, this structural equation modeling-based secondary analysis provides evidence that both school-based prevention efforts and social media use exert direct, quantifiable impacts on vaping behavior and harm perceptions of adolescents. The study’s findings support the development of an integrated prevention framework that bridges educational domains and digital strategies, utilizing evidence-based curricula, media literacy, and cross-sector collaboration to counteract pro-vaping influences and promote informed decision-making.

## Data Availability

Publicly available datasets were analyzed in this study. This data can be found at: https://www.floridahealth.gov/statistics-data/.
