# Correction: Social media exposure, school prevention, and adolescent vaping: a structural equation model-based secondary analysis of the Florida youth tobacco survey

**DOI:** 10.3389/fpubh.2026.1868375

**Published:** 2026-05-19

**Authors:** Martin Lange, Mary Martinasek, Nadine Heckert

**Affiliations:** 1 Department of Fitness & Health, IST University of Applied Sciences, Düsseldorf, Germany; 2 Department of Health Science and Human Performance, The University of Tampa, Tampa, FL, United States

**Keywords:** adolescents, risk perception, school prevention programs, social media, vaping

In the abstract, the first sentence “The increasing prevalence of vaping among adolescents raises public health concerns. Two factors influencing this trend are exposure to digital content on social media and school-based prevention programs.” was misleading. This has been corrected to read:

“Despite recent declines, the use of e-cigarette among adolescents remains the most popular. Two factors influencing vaping behavior are exposure to digital content on social media and school-based prevention programs.”

The statement that vaping increased is misleading: “The overall prevalence of vaping declined, while vaping gained popularity among those who use tobacco products.”

A correction has been made to the section **1 Introduction**, 1st paragraph, 3rd and 4th sentences:

“Electronic cigarettes [also known as e-cigarettes, vaping devices, or electronic nicotine delivery systems (ENDS)] are battery-powered devices that produce aerosols by heating liquids that typically contain flavoring agents, nicotine, and other chemicals (1). In 2024, a significant proportion of adolescents reported using e-cigarettes daily (26.3%), and 38.4% reported using them for at least 20 of the last 30 days (2). Despite a significantly decline in e-cigarette use among middle and high school students (2021: 2.07 million; 2024: 1.75 million), e-cigarettes remained the most popular tobacco product (3). The use of e-cigarettes in recent years has raised concerns about its long-term consequences and potential health risks (4, 5). According to longitudinal research, 45.5% of adolescents engaged in vaping, exceeding national predictions (5). The widespread use of e-cigarettes among adolescents has been linked to various risk factors, such as emotional abuse, substance use, and parental smoking, underscoring the need for early preventive measures (5–7).”

The reference for (3) was erroneously written as “Berry KM, Reynolds LM, Collins JM, Siegel MB, Fetterman JL, Hamburg NM, et al. E-cigarette initiation and associated changes in smoking cessation and reduction: the population assessment of tobacco and health study, 2013-2015. Tob Control. (2019) 28:42–9. doi: 10.1136/tobaccocontrol-2017-054108”. It should be “Shiva Shankar B. Forecasting youth tobacco use with the National Youth Tobacco Survey Data (2021-2024): implications for dental public health and cessation counseling in the United States. *Cureus*. (2025) 17:e86851. doi: 10.7759/cureus.86851”.

The original version of this article has been updated.

