# Supplementary material for: Social media exposure, school prevention, and adolescent vaping: a structural equation model-based secondary analysis of the Florida youth tobacco survey
Source: Front Public Health. 2026 Apr 9;14:1738207. doi: 10.3389/fpubh.2026.1738207 (PMC13102572; doi:10.3389/fpubh.2026.1738207)
Supplement: Supplementary file 1 [file Table_1.docx]

Supplementary Material

**Standardized Reporting** **Of Secondary Data Analyses (STROSA-1 Checklist)**

| Nr. | Items | **To Find in Line** |
| --- | --- | --- |
| Title, Abstract, Keywords | |  |
|  | Title and Abstract | 9-29 |
| Introduction | |  |
|  | Background and Rationale | 31-242 |
|  | Objectives | 85-87 |
| Method | | |
|  | Study Design | 245-253 |
|  | Frame | 246-247 |
|  | Legal Basis | 248-249 |
|  | Data Flow | 260-267 |
|  | Study Plan | n/a |
|  | Units of Analysis | 269-281 |
|  | Participants | 263-265 |
|  | Internal Validation | 279-281 |
|  | Variables | 284-301 |
|  | Classification System | n/a |
|  | Bias | n/a |
|  | Study size | 277-278 |
|  | Quantitative Methods | n/a |
|  | Statistical Analysis | 307-337 |
| Results | |  |
|  | Selection of the study population | n/a |
|  | Description of participants | 340-351 |
|  | Statistical measures | n/a |
|  | Main Results | 354-416 |
|  | Further Evaluation | n/a |
| Discussion | |  |
|  | Main Results | 419-427 |
|  | Limitations | 429-478 |
|  | Strengths | 480-500 |
|  | Interpretation | 502-600 |
|  | Transferability (and Practical Implications) | 602-670 |
| Further information | |  |
|  | Financing | 706 |
|  | Role of data owners | 698-704 |

n/a – not applicable
